# Emission wavelength tuning of fluorescence by fine structural control of optical metamaterials with Fano resonance

**DOI:** 10.1038/srep33208

**Published:** 2016-09-13

**Authors:** Y. Moritake, Y. Kanamori, K. Hane

**Affiliations:** 1 Department of Nanomechanics, Tohoku University, Japan

## Abstract

We demonstrated fine emission wavelength tuning of quantum dot (QD) fluorescence by fine structural control of optical metamaterials with Fano resonance. An asymmetric-double-bar (ADB), which was composed of only two bars with slightly different bar lengths, was used to obtain Fano resonance in the optical region. By changing the short bar length of ADB structures with high dimensional accuracy in the order of 10 nm, resonant wavelengths of Fano resonance were controlled from 1296 to 1416 nm. Fluorescence of QDs embedded in a polymer layer on ADB metamaterials were modified due to coupling to Fano resonance and fine tuning from 1350 to 1376 nm was observed. Wavelength tuning of modified fluorescence was reproduced by analysis using absorption peaks of Fano resonance. Tuning range of modified fluorescence became narrow, which was interpreted by a simple Gaussian model and resulted from comparable FWHM in QD fluorescence and Fano resonant peaks. The results will help the design and fabrication of metamaterial devices with fluorophores such as light sources and biomarkers.

Fluorescence control on quantum emitters is one of the important subjects for light emitting devices^1^,^2^, single photon source in quantum information technologies^3^, and biomarkers in the life science^4^. Recent developments in nanophotonics and fabrication technologies have enabled manipulation of fluorescence by using artificial structures such as micro cavities^5^, photonic crystals^6^, and metal structures^7^. Metamaterials open novel opportunities to control emission from quantum emitters by artificial structures^8^,^9^,^10^,^11^,^12^,^13^,^14^.

Metamaterials with Fano resonance, which is an interference effect between a bright mode and a dark mode with small radiative loss, is one of the promising candidates for fluorescence manipulation due to high quality factors (Q-factors). So far, Tanaka *et al*.^10^ have reported coupling between quantum dots (QDs) and Fano resonance in plasmonic metamaterials, in which enhanced fluorescence due to Purcell effect^15^ has been controlled by changing the periods of unit cell structures.

In this study, we demonstrate tuning of fluorescence emission wavelengths of QDs by fine structural control of optical metamaterials with Fano resonance. Wavelength tuning is essential for designing metamaterial devices. By changing the unit cell structures with high dimensional accuracy in the order of 10 nm, resonant wavelengths of Fano resonance are controlled. Coupling between Fano resonance and QDs leads to fine tuning of emission wavelengths of modified fluorescence.

## Results

An asymmetric double bar (ADB)^16^,^17^,^18^,^19^,^20^,^21^, which is composed of two gold bars having slightly different lengths, is employed to obtain Fano resonance in optical metamaterials. Asymmetry of the ADB structures makes a quadrupole mode excitable and interference between dipole and quadrupole modes leads to sharp Fano resonance. ADB metamaterials are one of the simplest structures showing Fano resonance and favorable to achieve Fano resonance in the optical region. Since excitation energy of a quadrupole mode in Fano resonance depends on an effective bar length of ADB structures, tuning of Fano resonance in ADB metamaterials can be achieved by controlling the shorter bar lengths.

**Figure 1:**
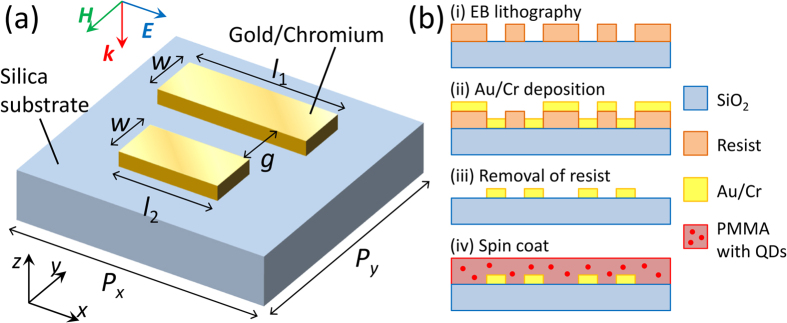
ADB metamaterial structure and fabrication processes. (**a**) A unit cell structure of ADB metamaterials. (**b**) Fabrication processes of ADB metamaterials covered with a PMMA layer containing QDs.

Figure 1(a) shows a unit cell structure of ADB metamaterials. The ADBs are arrayed periodically on a silica substrate with periods *P* = *P*_*x*_ = *P*_*y*_ of 450 nm. A gap *g* and a width *w* are both 90 nm. A long bar length *l*_1_ is fixed to be 268 nm while a short bar length *l*_2_ is varied from 268 nm to 186 nm in order to tune the Fano resonant wavelengths. Here, we define a difference between the two bars Δ*l* as *l*_1_−*l*_2_. A thickness of gold bars is 40 nm. An 800-nm-thick poly methyl methacrylate (PMMA) polymer containing QDs (the emission wavelength: 1366 nm, FWHM: 130 nm) covers the ADB metamaterials. The structural size of the unit cell should be designed for a resonant wavelength of Fano resonance to be coincident with an emission wavelength of QDs in the PMMA layer. Since a resonant wavelength of Fano resonance becomes 1.22 times longer after coating of the PMMA layer, an initial resonant wavelength of the ADB metamaterials without the PMMA layer was tuned to be a smaller wavelength than an emission wavelength of QDs.

**Figure 2:**
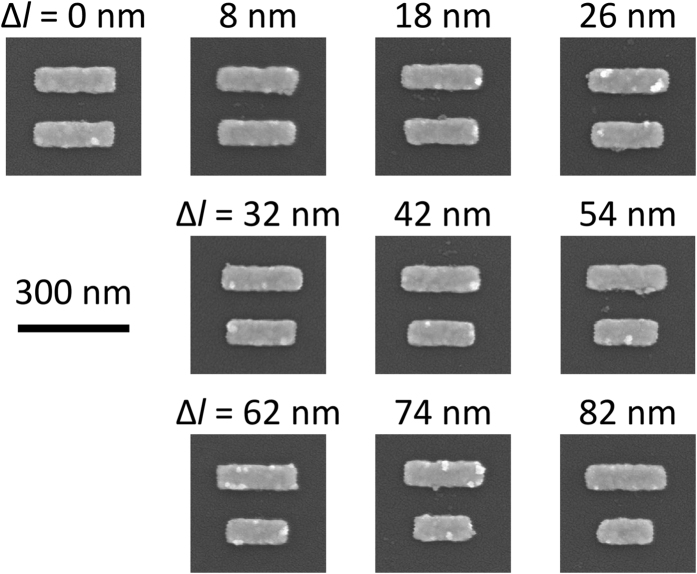
Fabrication results. SEM images of fabricated ADB metamaterials with various Δ*l*.

Fabrication was performed by a lift-off method and a spin coating technique as shown in Fig. 1(b). First, ADB metamaterials were patterned on an electron beam (EB) resist polymer using an EB lithography system, followed by EB deposition of chromium and gold thin films with thicknesses of 1 and 40 nm, respectively. Here, the chromium layer was used as an adhesion layer. Next, the EB resist polymer was removed and structures were formed on a silica substrate. Scanning electron microscope (SEM) images of fabricated ADB structures with various Δ*l* are shown in Fig. 2. The short bar lengths are successfully controlled in the order of 10 nm. Finally, toluene solution containing PMMA (5.0% in mass ratio) and PbS QDs (0.19% in mass ratio) was spin coated on ADB metamaterials. Rotational speed and time of spin-coating were 2000 rpm and 30 s, respectively.

**Figure 3:**
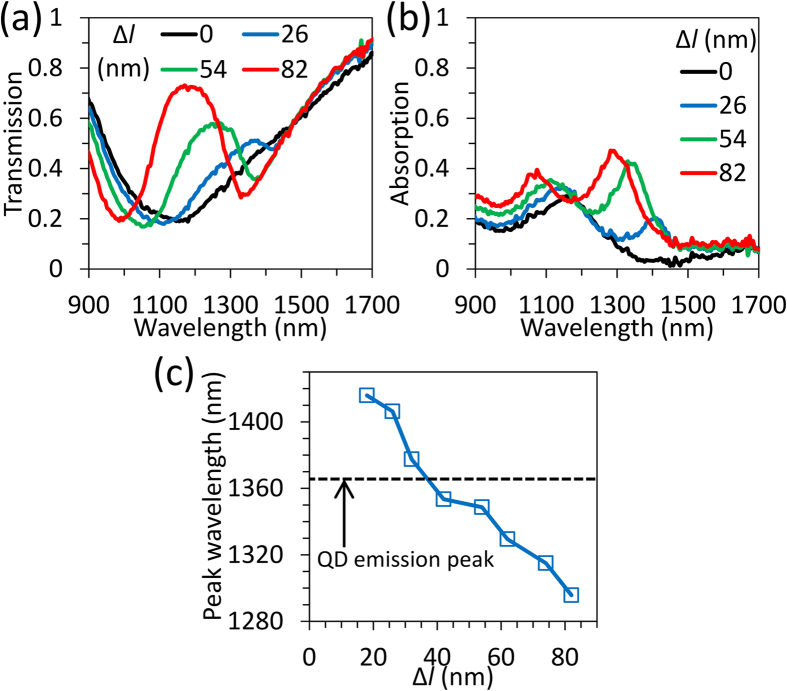
Results of optical measurements. Measured (**a**) transmission and (**b**) absorption spectra of the fabricated ADB metamaterials covered with the PMMA layer. (**c**) Peak wavelengths of Fano resonance as a function of Δ*l*.

Figure 3(a,b) show measured transmission and absorption spectra of the fabricated ADB metamaterials covered with a PMMA polymer in the case of Δ*l* = 0, 26, 54, and 82 nm, respectively. Here, absorption spectra (*A*) were calculated from transmission (*T*) and reflection (*R*) spectra by an expression (*A* = 1−*T*−*R*). An incident light is impinged normally to the surface of ADB metamaterials. Polarization of the incident light is along the bars (*x*-axis in Fig. 1(a)). In the case of Δ*l* = 0 nm, as shown in Fig. 3(a), only dipole resonance is observed as a dip at wavelengths around 1150 nm. On the other hand, in the case of Δ*l* = 26, 54, and 82 nm, Fano resonance is observed at wavelengths around 1350 nm which is close to an emission wavelength of QDs. Fano resonance also appears as peaks in absorption spectra as shown in Fig. 3(b). Figure 3(c) shows resonant wavelengths of Fano resonance are tuned from 1296 to 1416 nm by controlling Δ*l*, which is due to change in excitation energy of a quadrupole mode in Fano resonance with change in effective bar lengths in the ADB^17^,^18^.

**Figure 4:**
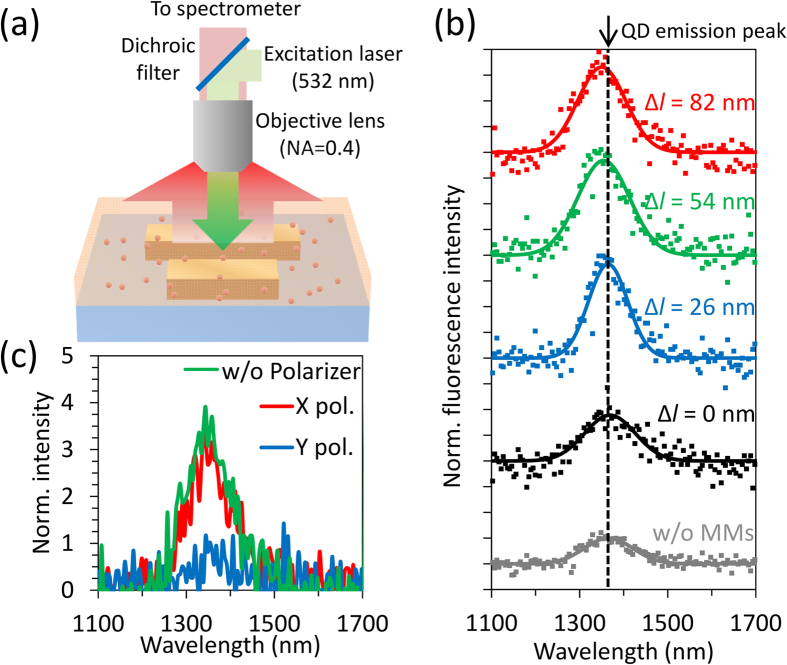
Results of measurements of fluorescence spectra. (**a**) The configuration of fluorescence measurements of QDs in a PMMA layer. (**b**) Measured fluorescence spectra of QDs hybridized with the ADB metamaterials. (**c**) Polarization dependence of fluorescence in the case of Δ*l* = 82 nm.

Fluorescence measurements were carried out using a standard microscopic fluorescence measurement system^22^. An excitation laser (wavelength: 532 nm, power: 10 mW) was reflected by a dichroic filter and focused on the ADB metamaterials with an objective lens (NA = 0.42) from a PMMA layer side. Fluorescence was collected by the same objective and passing through the dichroic filter, followed by detection using a spectrometer as shown in Fig. 4(a). Figure 4(b) shows results of fluorescence measurements. Gray squares show a fluorescence spectrum for QDs in the PMMA layer without the metamaterials. Black, blue, green, and red squares correspond to fluorescence spectra in the case of Δ*l* = 0, 26, 54, and 82 nm, respectively. Measured fluorescence spectra are normalized with the peak value of the fluorescence spectrum of QDs without ADB metamaterials. Solid lines in Fig. 4(b) are fitted curves by Gaussian functions. Fluorescence is enhanced about twice in the case of Δ*l* = 0 nm. In the case of Δ*l* = 26, 54, and 82 nm, moreover, approximately fourfold enhancement is observed. Figure 4(c) shows polarization dependence of fluorescence for Δ*l* = 82 nm. Polarization dependence was measured by positioning a polarizer between the dichroic filter and the spectrometer. Red and blue lines correspond to fluorescence when polarizing axes of the polarizer are set to along *x*- and *y*-axes (defined in Fig. 1(a)), respectively. A green line shows fluorescence measured without the polarizer. Strongly polarized enhanced fluorescence along the bars (*x*-axis) is observed for ADB metamaterials with Fano resonance. Such strong polarization dependence wasn’t observed in the case of Δ*l* = 0 nm.

## Discussion

We argue that observed modified fluorescence results from coupling between QDs and Fano resonance in metamaterials because enhancement rate and polarization dependence dramatically change depending on presence of Fano resonance in ADB metamaterials. Observed modification of fluorescence cannot be explained by simple convolution of QD fluorescence and reflection of metamaterials. Although reflection from metamaterials without Fano resonance (Δ*l* = 0 nm) was higher than the others, relatively low enhancement was observed. And modified fluorescence in the case of Δ*l* = 0 nm should have polarization dependence if metamaterials behave as wavelength selective mirrors.

When the coupling occurs, metamaterial structures can be considered as optical cavities at Fano resonance. Since quadrupole modes have relatively high Q-factors due to small radiative loss, energy from QDs can be stored and resonate in metamaterial structures. Therefore, QD fluorescence was enhanced and modified by optical cavities of metamaterials.

Although observed enhancement by Fano resonance was large compared with metamaterials without Fano resonance, our results do not indicate that Fano resonance can realize larger enhancement than dipole modes. In our experiments, dipole modes cannot effectively couple to emitters due to large mismatch between the resonant wavelengths (around 1150 nm) and the emission wavelength of QDs (1366 nm). Therefore, observed enhancement by dipole modes was relatively low.

**Figure 5:**
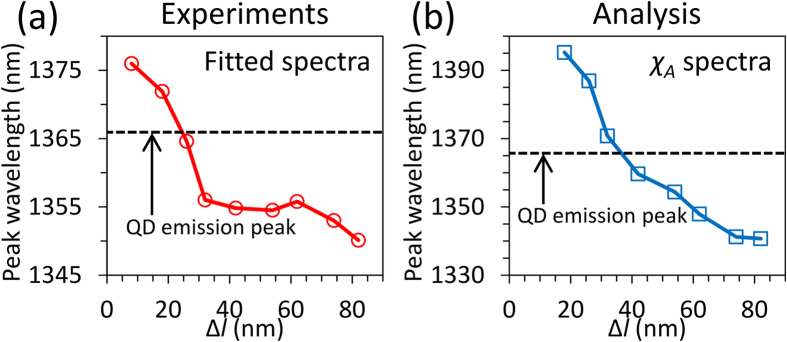
Tuning range of fluorescence emission peaks. Change in peak wavelengths of (**a**) fitted spectra and (**b**) *χ*_*A*_ spectra as a function of Δ*l*.

Figure 5 shows emission peak wavelengths of modified fluorescence extracted from the fitted spectra shown in Fig. 4(b). Peak wavelengths are tuned from 1350 to 1376 nm due to change in resonant wavelength of Fano resonance by controlling Δ*l* in ADB metamaterials. The tuning range of emission peak wavelengths becomes narrow compared with the tuning range of Fano resonance (1296~1416 nm) as indicated in Fig. 3(c).

To explain narrowing of the tuning range, we employed the analytical method introduced by Tanaka *et al*.^10^ Shapes of fluorescence spectra modified by ADB metamaterials are expected to be proportional to *χ*_A_(*λ*) = *FL*_0_(*λ*) × Δ*A*(*λ*). *FL*_0_(*λ*) is the fitted spectrum of QD fluorescence without metamaterials (gray line in Fig. 4(b)). Δ*A*(*λ*) is difference absorption spectra between the symmetric structure (Δ*l* = 0 nm) and the asymmetric structures (Δ*l* ≠ 0 nm). Δ*A*(*λ*) is used to extract the absorption contributed by only Fano resonance. The peak wavelengths extracted from *χ*_A_(*λ*) are shown in Fig. 5(b). The peak wavelengths of *χ*_A_(*λ*) vary from 1341 to 1395 nm and the analysis using absorption spectra successfully reproduces the narrowing of the tuning range, which also supports the coupling between QDs and Fano resonance. The deviation of tuning range between fitted spectra Fig. 5(a) and *χ*_A_(*λ*) spectra (Fig. 5(b)) is considered as results from undesired contributions of QDs in an uncoupling condition due to the relatively thick PMMA layer. Since QDs dispersing in PMMA farther than several hundred nanometers from metamaterial structures cannot couple to plasmonic modes in ADB metamaterials, modification of fluorescence doesn’t occur. Therefore, measured modified fluorescence consists of contributions from QDs both in coupling and uncoupling conditions.

Narrowing of the tuning range is resulted from the fact that FWHM of Fano resonance is comparable to that of original fluorescence spectra of QDs. Because the peak shapes of *FL*_0_(*λ*) and Δ*A*(*λ*) can be written by Gaussian functions, *χ*_*A*_(*λ)* can be represented by the product of two Gaussian functions. Therefore, *FL*_0_(*λ*) and Δ*A*(*λ*) are given as *FL*_0_(*λ*) = exp[ − (*λ*−*λ*_QD_)^2^/(2*σ*_1_^2^)] and Δ*A*(*λ*) = *A*_0_exp[ − (*λ*−*λ*_Fano_)^2^/(2*σ*_2_^2^)], where *λ*_QD_ is a peak wavelength of QD fluorescence, *A*_0_ is the peak value of Δ*A*(*λ*), *σ*_*i*_ (*i* = 1, 2) is given by *σ*_*i*_^2^ = Δ*λ*_*i*_^2^/(8ln2), Δ*λ*_1_ and Δ*λ*_2_ are FWHM for *FL*_0_(*λ*) and Δ*A*(*λ*), respectively. After multiplying the two Gaussian functions, we obtain expressions for the peak wavelengths *λ*_*χ*_ as follow,


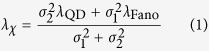


In the case of optical micro cavities with narrow FWHM (Δ*λ*_1_ ≫ Δ*λ*_2_), for example, whispering gallery or photonic crystals, Eq. (1) is deduced to be *λ*_*χ*_ = *λ*_Fano_, which means an emission peak wavelength of modified fluorescence corresponds to the resonant wavelength of the micro cavities. On the other hand, in the case of Fano resonance in optical metamaterials, FWHM of Fano resonance (~100 nm) is comparable to that of QD fluorescence (Δ*λ*_1_~Δ*λ*_2_). Therefore, Eq. (1) becomes *λ*_*χ*_ = (*λ*_QD_ + *λ*_Fano_)/2, and tuning range becomes narrow.

In summary, we experimentally demonstrated fine emission wavelength tuning of QD fluorescence by fine structural control of optical metamaterials with Fano resonance. ADB metamaterials were fabricated by a lift-off method. Resonant wavelengths of Fano resonance were controlled from 1296 to 1416 nm by changing the short bar lengths of ADB structures with high dimensional accuracy in the order of 10 nm. Fluorescence of QDs embedded in a polymer layer on ADB metamaterials was modified due to coupling to Fano resonance, resulting in four-fold enhancement, strong polarization dependence along the bars, and fine tuning from 1350 to 1376 nm. Analysis using absorption peaks of Fano resonance reproduced narrowing of the tuning range. The expression using Gaussian functions revealed that narrowing of the tuning range was resulted from a comparable FWHM in Fano resonance and original fluorescence spectra of QDs. The results will help the design and fabrication of metamaterial devices with fluorophores such as light sources and biomarkers.

## Additional Information

**How to cite this article**: Moritake, Y. *et al*. Emission wavelength tuning of fluorescence by fine structural control of optical metamaterials with Fano resonance. *Sci. Rep.* **6**, 33208; doi: 10.1038/srep33208 (2016).
